# Is ^70^Zn(d,x)^67^Cu the Best Way to Produce ^67^Cu for Medical Applications?

**DOI:** 10.3389/fmed.2021.674617

**Published:** 2021-07-05

**Authors:** Etienne Nigron, Arnaud Guertin, Ferid Haddad, Thomas Sounalet

**Affiliations:** ^1^Laboratory SUBATECH, CNRS/IN2P3, IMT Atlantique, Université de Nantes, Nantes, France; ^2^GIP ARRONAX, Saint Herblain, France

**Keywords:** ^67^Cu, production, theranostic, cross section, deuteron reactions, accelerators

## Abstract

The pair of copper radionuclides ^64^Cu/^67^Cu (T_1/2_ = 12. 7 h/61.8 h) allows, respectively, PET imaging and targeted beta therapy. An analysis of the different production routes of ^67^Cu with charged particles was performed and the reaction ^70^Zn(d,x) route was identified as a promising one. It may allow the production of ^67^Cu without ^64^Cu. The production cross section has been measured up to 28.7 MeV. Measurements were done using the well-known stacked-foils technique using 97.5% enriched ^70^Zn homemade electroplated targets. These measurements complement at higher incident energies the only set of data available in nuclear databases. The results show that using a 26 MeV deuteron beam and a highly enriched ^70^Zn target, it is possible to produce high purity ^67^Cu comparable to that obtained using photoproduction. This production route can be of interest for future linear accelerators under development where mA deuteron beams can be available if adequate targetry is developed.

## Introduction

^67^Cu (T_1/2_ = 61.8 h) is a radionuclide with physical properties convenient for therapeutic use as targeted radiotherapy. It is a beta-emitter with a maximum energy of 561 keV, which corresponds to an electron path of about 3 mm in water (1). Its energy range is comparable to that of the ^177^Lu currently used in targeted radiotherapy (2). ^67^Cu emits also photons of 184.6 keV (3) which offers the possibility of carrying out SPECT imaging. It can be used either prior the treatment as an imaging agent or during therapy to monitor the diffusion and distribution of the ^67^Cu radiolabelled radiopharmaceuticals.

To select the best production route both cross section data associated to the production of ^67^Cu and to contaminants are of primary importance. Among contaminants, coproduced copper isotopes are of great concern, as they cannot be removed from the final product by chemical separation. Especially ^64^Cu, with its 12.7 h half-life, will have an impact on the specific activity. It is then interesting to look at production routes that reduce or exclude the co-production of ^64^Cu, even if its real impact on the patient and staff needs to be studied and clarified.

The most cited production route for ^67^Cu production uses enriched ^68^Zn target bombarded by high-energy protons (4–7). Large quantities can be produced but it is not possible to limit ^64^Cu co-production. In the 90's and 2000's, the ^64^Ni(α,p)^67^Cu reaction with an ^64^Ni enriched target and an alpha beam was also studied. Experimental cross sections for this reaction are known with a maximum cross section value around 35 mb at 22 MeV (7, 8). The threshold energy for the production of ^64^Cu through ^64^Ni(α,n+t) is equal to 23.7 MeV. Using the very high enrichment level of ^64^Ni available, there is a possibility to produce ^67^Cu free of ^64^Cu by limiting the beam energy below this latter value. However, alpha beams are poorly available and thermal constrains associated to high intensity alpha beam are very penalizing.

An alternative consists to use an enriched ^70^Zn target bombarded with either protons or deuterons. In this case, the production of ^64^Cu can be limited by an appropriate choice of the beam energy and high target enrichment. As an example, the ^70^Zn(d,x)^64^Cu reaction threshold is 26.4 MeV whereas that for ^67^Cu production is 0 MeV (see Supplementary Table 1). ^70^Zn(p,α)^67^Cu reaction cross section reaches a maximum of 15 mb at 15 MeV (7, 9) whereas the available data for ^70^Zn(d,x)^67^Cu (10) show that the reaction cross section maximum is higher, even if the exact value is not known as this data set do not cover the whole energy range of interest.

In this work, we have measured ^70^Zn(d,x)^67^Cu production cross section up to 28.7 MeV in order to determine the position and value of the maximum. Production cross sections of contaminants have been also extracted. Using these new data, we were able to determine production yields and, with the help of TALYS 1.9 calculations (12), the expected specific activity of the final product.

## Materials and Methods

Production cross sections for the ^70^Zn(d,x)^67^Cu reaction was measured using the stacked-foils activation method (4, 13–16). A series of six irradiations, spread for over 7 months, was carried out at the GIP ARRONAX C70 cyclotron, Saint-Herblain, France. In our experiments, a stack was made of two patterns each composed of a 10 μm thick enriched ^70^Zn (97.5% purity) electroplated on a 25 μm Ni foil (99.9% purity) followed by an aluminum foil (10 μm, 99.0% purity). Their thicknesses were determined assuming homogeneity by weighing and performing surface calculation with a high definition scanner. The obtained values are reported in Supplementary Table 2. Aluminum is used as a foil to catch recoil nuclei. The stack was placed inside a dedicated vacuum chamber positioned at the end of the AX beam line. It contains an instrumented Faraday cup used to determine the particle flux going through the stack. We limited the total thickness of the stack to prevent a large geometrical straggling that will result on an increased uncertainty on the flux measurement. A Ti foil, having an area equivalent to the ^70^Zn deposit, was added between the Ni and Al foil of the second pattern to obtain a second independent flux value by measuring the production cross section associated to ^nat^Ti(d,x)^48^V. This reaction is well-known and is used as a reference (17) (monitor) to make sure everything went well during our experiments. After irradiation which stands for 1 h with an average current of ~50 nA, activities of each thin foils were measured using gamma spectroscopy (HPGe). The well-known activation formula was used to calculate the cross section values.

Gamma analyses were carried out using the FitzPeaks software (18). Spectra were recorded in a suitable geometry calibrated in energy and efficiency with standard ^57^Co, ^60^Co, and ^152^Eu sources from LEA-CERCA (France). The full widths at half maximum were 1.05 keV at 122 keV (^57^Co) and 1.97 keV at 1,332 keV (^60^Co). No activity was measured for recoil nuclei on catching foils. The ^48^V activity was measured only after full ^48^Sc decay, 3 weeks after End Of Beam (EOB).

The energy loss of the particles passing through the stack has been calculated from the equations of Ziegler et al., using their SRIM-2013 software (19). The energies are calculated in the middle of the foils and are shown in Supplementary Table 2.

Chemical preparations and electroplating were made on site using enriched ^70^Zn metallic powder from Trace Sciences International. The enrichment level of ^70^Zn was 97.5%, ^68^Zn 2.2%, ^67^Zn 0.1%, ^66^Zn 0.1% and ^64^Zn 0.1%. All solutions were freshly prepared with ultra-pure water treated with Milipore Milli Q system. The metallic powder was dissolved in diluted sulfuric acid (1 M) to obtain zinc sulfate, then evaporated to dryness and rinsed twice with ultra-pure water. For each preparation, pH was adjusted to two by addition of sulfuric acid. The electroplating was carried out in a simple homemade three-electrode Teflon cell. The counter electrode was made of platinum and an Ag-|AgCl|Cl^−^ (saturated KCl) electrode was used as reference and was connected to the cell. The deposition area was delimited during electroplating using a silicon gasket and corresponds to 4 cm^2^. Electroplating was performed by using the VoltaLab050 potentiostat. The deposition was obtained by applying a constant current density of −20 mA/cm^2^. During plating, the temperature was kept constant at 30°C and the solution was stirred at 300 rpm for homogenization purpose. To reach a thickness of 10 μm, a deposition time of 30 min was necessary.

The presence of ^68^Zn (2.2%) in the target material implies potential contamination, which is taken into account during the analysis. Indeed, the interaction of deuterons on ^68^Zn can produce ^67^Ga (E_threshold_ = 14.6 MeV) whereas ^67^Ga is not produced in our energy range by deuteron interactions with ^70^Zn (E_threshold_ = 30.8 MeV). ^67^Ga decays to ^67^Zn as ^67^Cu leading to common gamma rays during both decays, fortunately with different intensities. As an example, the 184 keV gamma ray corresponds to an intensity of 48.7% in ^67^Cu decay whereas it is only 21.41% for ^67^Ga decay (3). The same holds for the 300 keV gamma line which intensity is 0.797% for ^67^Cu and 16.64% for ^67^Ga. Therefore, we used this property to discriminate production of ^67^Cu and ^67^Ga. This is based on a set of equations (1−3) involving the number of gamma collected at 184 keV and 300 keV. These equations relate to the total number of gammas collected, N_TOT_, from a gamma peak to the number of gamma collected from each contributor.

(1) $$\begin{array}{l} N_{TOT}^{184}=N_{67Cu}^{184}+N_{67Ga}^{184}\\ N_{TOT}^{300}=N_{67Cu}^{300}+N_{67Ga}^{300} \end{array}$$

Equations can be written as:

(2) $$\begin{array}{l} N_{TOT}^{184}=k_{1}\;Act{(}^{67}Cu)+k_{2}\;Act{(}^{67}Ga)\\ N_{TOT}^{300}=k_{3}\;Act{(}^{67}Cu)+k_{4}\;Act{(}^{67}Ga)\\ \;With\;k_{i}^{x}=\frac{{\text{ε}}_{i}^{x}\;I_{i}^{x}\;(1\;-\;e^{-{\text{λ}}_{i}\;t_{LT}})}{{\text{λ}}_{i}} \end{array}$$

Where i corresponds to a specific radionuclide, × to a given gamma line, ε to the detector efficiency at this energy, I to the intensity of the gamma emission, λ to the radioactive constant and t_LT_ to the acquisition time. Expressed in terms of activity of each radionuclide, the system of equations is written as follows:

(3) $$\begin{array}{l} Act{(}^{67}Cu)=\frac{1}{k_{1}k_{4}-k_{2}k_{3}}(\;k_{4}\;N_{TOT}^{184}-\;k_{2}\;N_{TOT\;}^{300})\\ Act{(}^{67}Ga)=\frac{1}{k_{1}k_{4}-k_{2}k_{3}}(\;k_{1}\;N_{TOT}^{300}-\;k_{3}\;N_{TOT}^{184}\;) \end{array}$$

The activities of ^67^Cu and ^67^Ga are determined from equations in (3). The uncertainties associated with this activity calculation have been established according to the following equation:

(4) $$\sigma (Act)=\sum_{j}\left|\frac{\partial Act}{\partial y_{j}}\right|\;\sigma \;(y_{j})$$

Where y represents the different parameters involved in each equation (3).

## Results and Discussion

**Figure 1:**
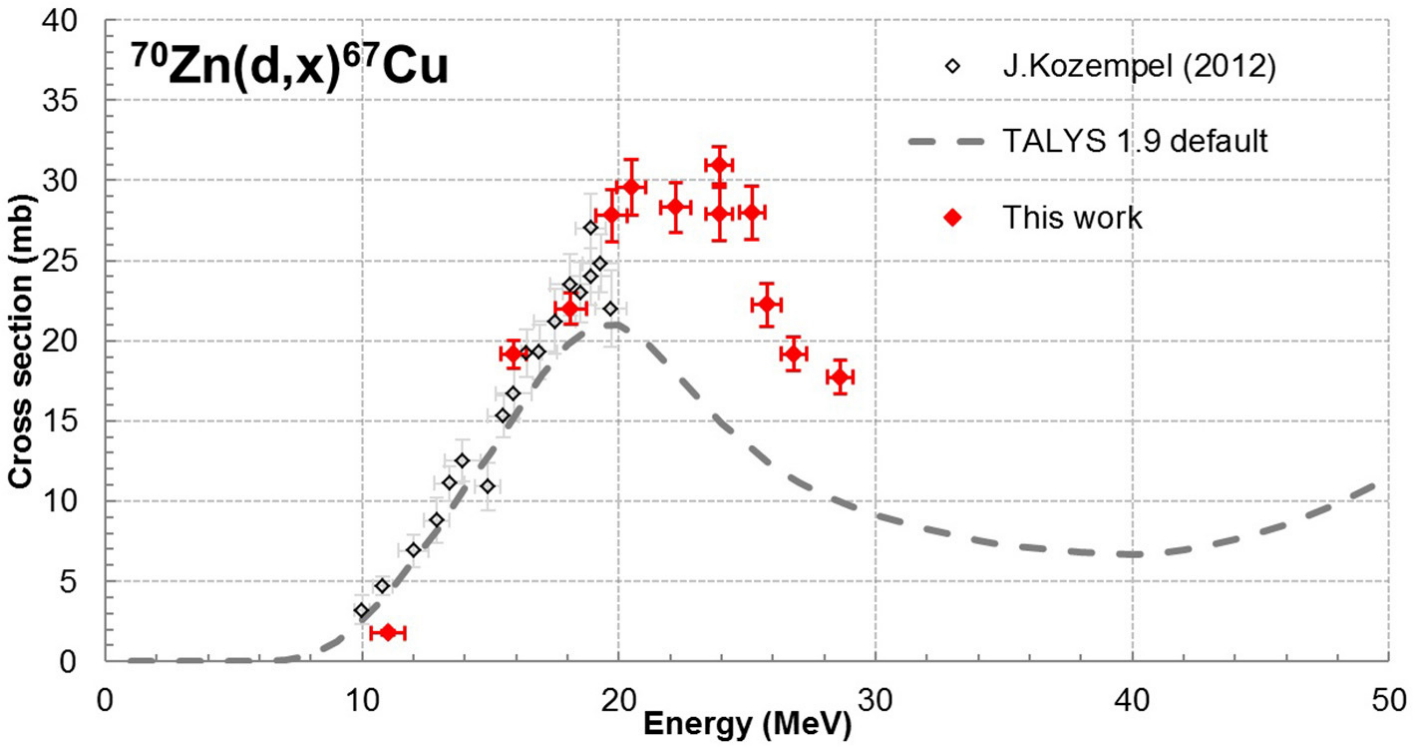
Cross sections of reaction ^70^Zn(d,x)^67^Cu (10).

**Figure 2:**
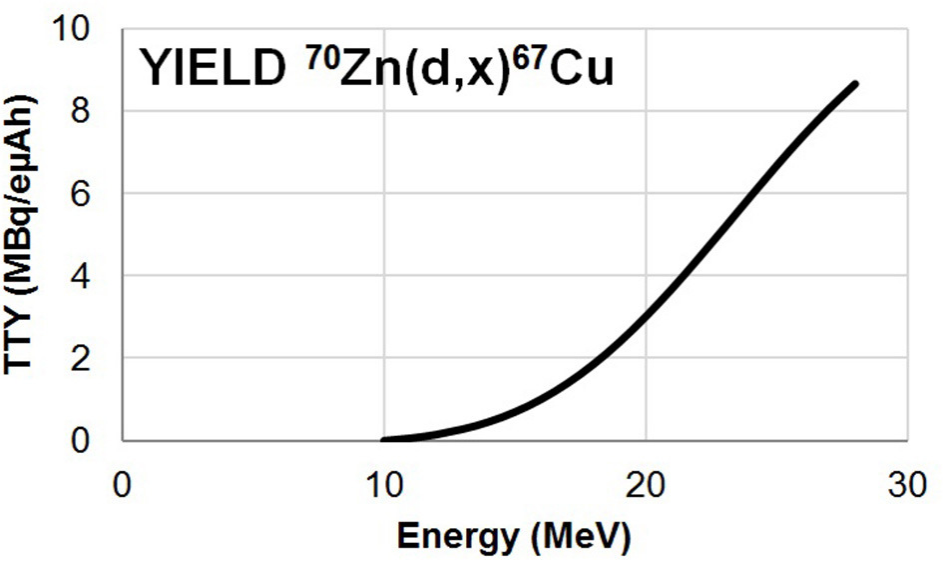
Thick Target Yield curve for the ^70^Zn(d,x)^67^Cu reaction (11).

**Figure 3:**
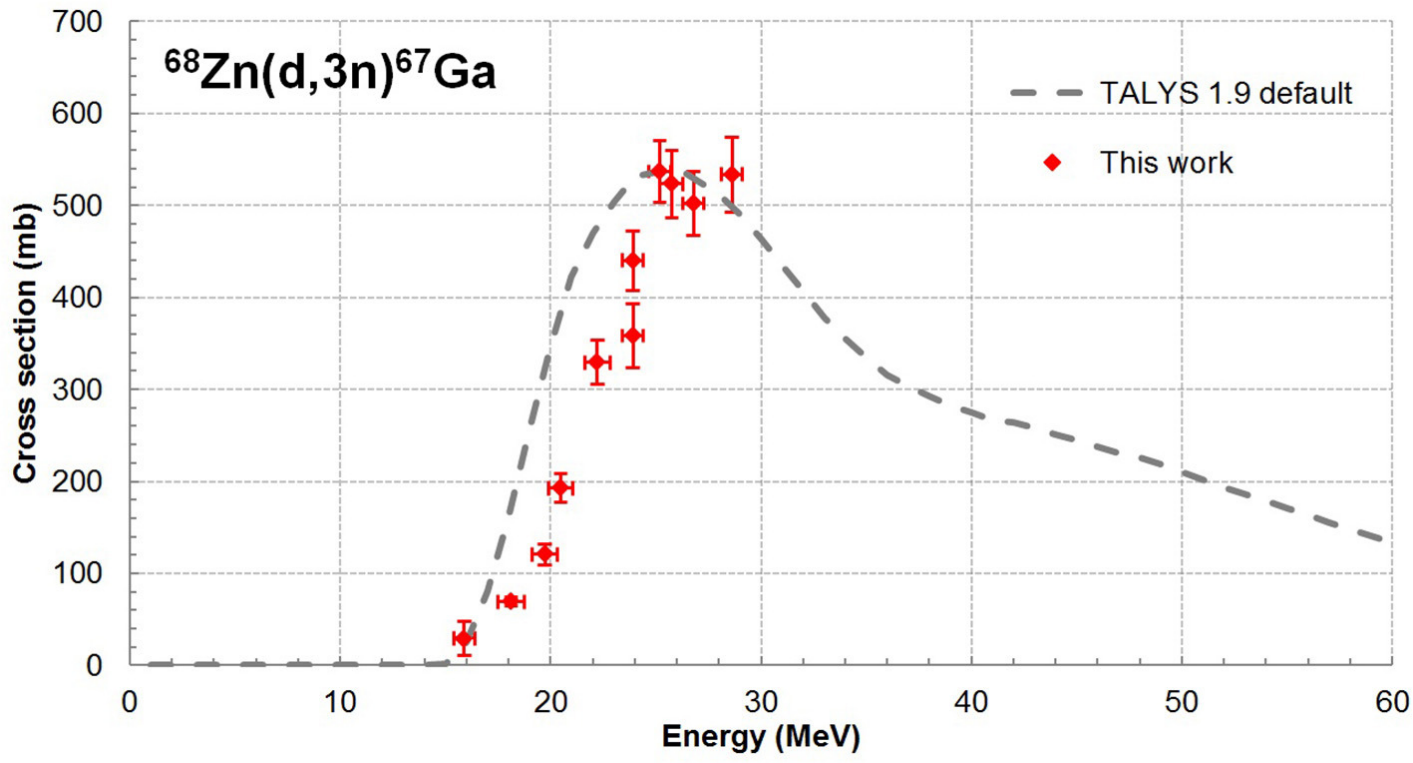
Cross sections of the ^68^Zn(d,3n)^67^Ga reaction.

In these experiments, excitation functions up to 28.7 MeV were measured for ^67^Cu and ^67^Ga from the zinc deposit whereas ^61^Cu, ^55, 56, 57, 58^Co were extracted from the Ni backing and ^48^V from the Ti foil. All nuclear reactions involved are reported in the Supplementary Table 3 as well as gamma lines used for the analysis of each radionuclide. Our results are displayed on Figures 1–3, in values reported in Table 1. The simulation code of nuclear reactions TALYS 1.9 was used to extend the study in particular on the stable (^65^Cu) nucleus production. This is the reason why, in addition to our data points, we have displayed on our figures the values obtained with TALYS 1.9 (12). Reactions on the Ni support and on the Ti foil are monitor reactions for which reference data exist at IAEA (17). Through these monitor cross section values, we can control the measurement of the particle flux and consequently the correct execution of the experiment.

**Table 1:** Cross section values measured in this study for reactions having taken place in zinc, nickel, and titanium.

**Energy (MeV)**	**Cross section (mb)**							
	**^**67**^Cu**	**^**67**^Ga**	**^**61**^Cu**	**^**55**^Co**	**^**56**^Co**	**^**57**^Co**	**^**58**^Co**	**^**48**^V**
9.8 ± 0.7	-	-	-	-	-	-	-	124.7 ± 4.0
10.4 ± 0.7	-	-	41.5 ± 2.0	0.3 ± 0.1	35.2 ± 1.1	3.0 ± 0.2	113.4 ± 3.5	-
11.0 ± 0.7	1.8 ± 0.1	-	-	-	-	-	-	-
15.4 ± 0.5	-	-	23.4 ± 1.8	10.1 ± 0.3	16.9 ± 0.6	28.7 ± 1.0	219.5 ± 6.9	-
15.9 ± 0.5	19.2 ± 0.9	29.3 ± 18.2	-	-	-	-	-	-
17.1 ± 0.7	-	-	-	-	-	-	-	333.2 ± 10.7
17.5 ± 0.6	-	-	21.1 ± 1.3	12.4 ± 0.4	12.4 ± 0.5	36.0 ± 1.3	212.9 ± 6.7	-
18.1 ± 0.6	22.0 ± 1.0	69.0 ± 4.8	-	-	-	-	-	-
18.9 ± 0.6	-	-	-	-	-	-	-	337.2 ± 13.0
19.3 ± 0.6	-	-	15.9 ± 1.1	14.9 ± 0.5	9.9 ± 0.4	56.7 ± 1.8	219.4 ± 6.9	-
19.7 ± 0.6	27.8 ± 1.6	120.7 ± 11.2	-	-	-	-	-	320.7 ± 9.9
20.0 ± 0.6	-	-	16.5 ± 2.8	18.6 ± 0.6	9.4 ± 0.4	88.3 ± 2.8	233.5 ± 7.3	-
20.5 ± 0.6	29.6 ± 1.7	192.5 ± 15.6	-	-	-	-	-	-
21.1 ± 0.6	-	-	-	-	-	-	-	270.1 ± 8.6
21.5 ± 0.6	-	-	17.0 ± 2.9	22.8 ± 0.5	8.1 ± 0.3	172.4 ± 3.7	221.6 ± 5.2	-
22.2 ± 0.6	28.3 ± 1.6	329.2 ± 24.2	-	-	-	-	-	-
23.5 ± 0.5	-	-	15.3 ± 0.5	23.4 ± 0.7	7.6 ± 0.4	230.1 ± 7.2	204.8 ± 6.4	-
23.5 ± 0.5	-	-	16.0 ± 1.5	23.5 ± 0.7	7.7 ± 0.4	235.6 ± 7.4	202.6 ± 6.4	-
23.9 ± 0.5	27.9 ± 1.7	440.0 ± 32.7	-	-	-	-	-	-
23.9 ± 0.5	30.9 ± 1.1	358.4 ± 34.5	-	-	-	-	-	-
24.8 ± 0.5	-	-	14.4 ± 1.0	22.2 ± 0.7	7.2 ± 0.3	262.7 ± 8.1	182.0 ± 5.8	-
25.2 ± 0.5	28.0 ± 1.7	537.8 ± 33.3	-	-	-	-	-	182.7 ± 5.7
25.4 + 0.5	-	-	-	22.7 ± 0.7	8.9 ± 0.4	334.0 ± 10.3	172.6 ± 5.4	-
25.8 ± 0.5	22.2 ± 1.3	523.3 ± 36.4	-	-	-	-	-	-
26.6 ± 0.5	-	-	17.0 ± 2.9	20.0 ± 0.4	10.6 ± 0.5	362.4 ± 7.8	156.5 ± 3.9	-
26.8 ± 0.5	19.2 ± 1.1	501.9 ± 34.5	-	-	-	-	-	-
28.2 ± 0.5	-	-	23.9 ± 11.2	17.8 ± 0.6	13.2 ± 0.5	414.0 ± 12.8	135.2 ± 4.3	-
28.6 ± 0.5	17.7 ± 1.1	533.5 ± 41.0	-	-	-	-	-	-

### The ^nat^Ti(d,x)^48^V Reaction

^48^V (T_1/2_ = 15.973 d) is produced by the ^nat^Ti(d,x)^48^V reaction. The foil has been cut and positioned to have the same surface area as the ^70^Zn deposit. If the entire beam does not pass through the deposit because it is too wide, this will also be the case for the titanium foil. In this case, the extracted values will not be in agreement with the monitor cross section.

During the irradiation of a titanium foil, not only ^48^V is produced but also ^48^Sc which decay to the same daughter nucleus than ^48^V. To get rid of ^48^Sc, we let it decay, at least 19 days, until the vast majority of the ^48^Sc disintegrates. The results are presented in Supplementary Figure 1.

These data are in agreement with experimental values available in the literature (20–25). The agreement of these data shows that the foils and deposits were crossed by the entire beam. The agreement is generally good with the cross section recommended by the IAEA (13). In agreement with experimental data in the literature, our points indicate a peak around 19 MeV which is not described by the IAEA curve.

### The ^nat^Ni(d,x)^61^Cu Reaction

The ^61^Cu (T_1/2_ = 3.339 h) is produced by the ^nat^Ni(d,x) reaction. The gamma emissions used for activity measurement are 282.956 keV (12.2%), 373.050 keV (2.15%), 588.605 keV (1.17%), 656.008 keV (10.77%), 908.631 keV (1.102%), and 1185.234 keV (3.75%). Ni is the backing of the Zn target. This reaction is a monitor reaction for which the IAEA proposes a reference curve. Our data are presented in Supplementary Figure 2. One data point shows large error bar. This is due to a late counting that induces a lack of statistics. However, our data are in good agreement with experimental data available in the literature (20–22, 26–31) and with the IAEA curve (17). This confirms that the experiment was well-controlled.

### The ^70^Zn(d,x)^67^Cu Reaction

^67^Cu (T_1/2_ = 61.83 h) cross sections were determined from gamma emissions at 184.6 keV (48.7%) and 300.2 keV (0.797%) and Equation 3. The production contribution of ^67^Cu from ^68^Zn (present at 2.2%) is not taken into account as it is expected to be negligible [of the order of 1 mb in the model calculation TALYS 1.9 (12)]. Our data are presented in Figure 1.

Our data complement the data already presented in the literature (10) to higher incident energies. They are in good agreement with Kozempel et al. (10) and allow determining the energy of the maximum of the cross section near 23 MeV and its value around 30 mb. This information will allow to more precisely defining optimal beam parameters for ^67^Cu production using a deuteron beam and a ^70^Zn target.

The TALYS 1.9 simulation code was used with its default set of parameters to calculate the cross section of the ^70^Zn(d,x)^67^Cu reaction. The code calculation is not able to describe the data. There is a slight shift toward lower energies of the maximum and the data are underestimated by the code calculation.

Using our dataset and that of Kozempel et al., we have performed a ^67^Cu thick target yield calculation (TTY) according to (32). In the formula (5), σ is the production cross section, H is the enrichment and the purity of the foil, N_a_ is the Avogadro's number, λ is the decay constant of the radioisotope, Z is the charge of the fully ionized projectile, e is the elementary charge, M is the atomic mass of the target, E_max_ and E_min_ are the maximal and minimal energy of the projectile penetrating the target and dE/dx is the stopping power of the projectile in the irradiated target. The result is plotted on Figure 2 as a function of the incident deuteron energy.

(5) $$TTY\;\left(E\right)=\frac{H\;N_{a\;}\text{λ}}{Z\;e\;M}\int_{Emin}^{Emax}\frac{\sigma (E)}{dE/dx(E)}\;dE$$

We can clearly see that the yield increases more rapidly around the maximum as expected. Taking into account the threshold of 26.4 MeV associated to the production of ^64^Cu, the shape of the ^67^Cu cross section and the high price of ^70^Zn, the preferred energy range for production through this route is 16–26 MeV. This energy range corresponds to a ^70^Zn thickness of 576 μm.

By setting the beam intensity at 1 μA, 1 h of irradiation and a target purity of 97.5%, the estimated activity produced over the 16–26 MeV energy range is 6.2 MBq. This result is higher than that of Hosseini et al. (33) which corresponds to model calculations. The main difference comes from the cross section values used in this study that are lower than those experimental ones.

Interesting information is related to the expected specific activity of the final product. To determine the contribution of each copper isotope, experimental data were used for ^67^Cu and TALYS 1.9 calculations using default parameters for the other isotopes.

With 80 μA and 40 h of irradiation, the expected activity of ^67^Cu EOB is 16.4 GBq and the ^67^Cu represents only 35.77% of the total copper activity due to the production of short-lived ^66, 68, 69^Cu (T_1/2_ : 5.12 min; 0.515 min; 2.85 min). However, by waiting 70 min after irradiation for decays, the activity of ^67^Cu reaches 99.99% of the total copper activity and, at this time, the specific activity is 1.87 × 10^3^ MBq/nmol or 2.79 × 10^4^ GBq/mg. This specific activity value is very close to the theoretical maximum (2.80 × 10^4^ GBq/mg). This small difference is due to the production of ^65^Cu.

Using an enriched target such as the one used in this study (97.5%), ^67^Cu represents 99.99% of the copper activity after 121 h of decay and the specific activity is 2.52 × 10^4^ GBq/mg (99.00% reached for 15 h of decay). This is due to the production of ^64^Cu in a thick target containing a non-negligible proportion of ^68^Zn. Using a higher enrichment will reduce the impact of other copper isotopes and especially ^64^Cu. However, during this 15 h decay time, the copper extraction chemistry can be performed as well as the sample delivery.

### The ^68^Zn(d,x)^67^Ga Reaction

In our experimental condition, ^67^Ga (T_1/2_ = 78.3 h) is produced only from the residual amount of ^68^Zn through ^68^Zn(d,3n) reaction. Indeed, the energy threshold for the production of ^67^Ga using ^70^Zn is equal to 30.77 MeV. As ^67^Ga decays to the same daughter nuclide as ^67^Cu, its contribution in the spectra was extracted from equations (3) using gamma emissions of 184.6 keV (21.41%) and 300.2 keV (16.64%). Cross sections data for the ^68^Zn(d,3n)^67^Ga reaction are shown in Figure 3 as red dots. There is no data for this reaction in the literature. The only possibility is to compare to calculated values using the TALYS 1.9 code with the default set of parameters (dashed line). The amplitude of the cross section is compatible with the data. Additional data at higher energies will help to constrain the theoretical models contained in the simulation code. The cross section is relatively high which implies, despite a ^68^Zn concentration of 2.2%, a non-negligible activity production of ^67^Ga. The percentage of ^67^Ga in the total ^67^Cu+^67^Ga activity EOB varies from 2.4% to 31.8% with the minimum at 15.9 MeV and the maximum at 26.8 MeV which follow the minimum and maximum of the cross section curve, Figure 3.

### The ^64^Cu Production

The ^64^Cu (T_1/2_ = 12.701 h) emits a gamma of 1345.77 keV at 0.475% during its beta+ decay to 61.5%. Due to the low emission intensity, it was not detected. Moreover, its production is possible on several isotopes present in the target (^68^Zn and ^70^Zn) and with nickel support (^62^Ni and ^64^Ni) which do not allow unambiguous identification of its origin. Therefore, the calculation of the cross section of a specific reaction could not be done. So, no cross section values for ^64^Cu are presented.

## Discussion

In this work, we have determined the ^67^Cu production cross section associated to the use of a deuteron beam impinging an enriched ^70^Zn target. This production route is of great interest as it limits strongly the production of ^64^Cu that is directly linked to the level of ^68^Zn impurity in the target. In our study, data up to 28.7 MeV have been obtained using the stacked-foils technique. Beam intensity has been obtained using an instrumented Faraday cup. Cross sections for the following monitor reaction ^nat^Ti(d,x)^48^V, ^nat^Ni(d,x)^56^Co, ^nat^Ni(d,x)^56^Co, and ^nat^Ni(d,x)^61^Cu have been extracted from the target backing and the Ti monitor foil. These experimental values are in agreement with datasets available in the literature indicating that the experiment was well-controlled. Our new data on ^70^Zn(d,x)^67^Cu allows to clearly identifying the maximum of the cross section around 30 mb for an incident energy of 23 MeV. Based on these data, we propose to use a deuteron beam of 26 MeV and a target of 576 μm (leading to outgoing deuteron energy of 16 MeV) as optimum irradiation parameters. This leads to a production yield of 6.4 MBq/μA/h and allows the production of 16.4 GBq with a specific activity of 2.79 × 10^4^ GBq/mg for an irradiation of 40 h with an intensity of 80 μA followed by a decay period of 70 min and with a 100% enriched ^70^Zn target. These amounts of ^67^Cu activity produced with high specific activity especially without the presence of ^64^Cu are suitable for clinical studies. This makes the ^70^Zn(d,x) an attractive production route for ^67^Cu. It can become the production route of choice only if the use of linear accelerators such as SPIRAL2 (34) or SARAF (11) is set-up that will provide beam intensities in the mA range and if adequate targetry is developed.

## Data Availability Statement

The raw data supporting the conclusions of this article will be made available by the authors, without undue reservation.

## Author Contributions

EN: main contributor (experiment, analyses, and article writing). AG: experiment and article review. FH: project leader and article review. TS: experiment, target manufacturing, and article review. All authors contributed to the article and approved the submitted version.

## Conflict of Interest

The authors declare that the research was conducted in the absence of any commercial or financial relationships that could be construed as a potential conflict of interest.
