# Supplementary material for: Is 70Zn(d,x)67Cu the Best Way to Produce 67Cu for Medical Applications?
Source: Front Med (Lausanne). 2021 Jul 5;8:674617. doi: 10.3389/fmed.2021.674617 (PMC8287065; doi:10.3389/fmed.2021.674617)
Supplement: Supplementary file 1 [file Table_1.docx]

Table S1 - Threshold energies of reactions with deuterons producing copper isotopes for different monoisotopic enriched zinc targets.

| Reaction | Energy threshold (MeV) | | | | |
| --- | --- | --- | --- | --- | --- |
|  | ^63^Cu | ^64^Cu | ^65^Cu | ^67^Cu | |
| ^67^Zn(d,x) | 7.9 | 0 | 0 | | 2.1 |
| ^68^Zn(d,x) | 18.5 | 10.3 | 0.1 | | 4.6 |
| ^70^Zn(d,x) | 34.6 | 26.4 | 16.2 | | 0 |
