# Supplementary material for: Is 70Zn(d,x)67Cu the Best Way to Produce 67Cu for Medical Applications?
Source: Front Med (Lausanne). 2021 Jul 5;8:674617. doi: 10.3389/fmed.2021.674617 (PMC8287065; doi:10.3389/fmed.2021.674617)
Supplement: Supplementary file 2 [file Table_2.docx]

Table S2 - Calculated foil thicknesses and deuteron energy in the middle of each foil.

| Energy (MeV) | Thickness (µm) | | | |
| --- | --- | --- | --- | --- |
|  | ^70^Zn | Ni | Ti |  |
| 9.8 ± 0.7 | - | - | 10.00 ± 0.08 |  |
| 10.4 ± 0.7 | - | 25.97 ± 0.09 | - |  |
| 11.0 ± 0.7 | 6.57 ± 0.03 | - | - |  |
| 15.4 ± 0.5 | - | 26.25 ± 0.09 | - |  |
| 15.9 ± 0.5 | 7.88 ± 0.04 | - | - |  |
| 17.1 ± 0.7 | - | - | 9.61 ± 0.08 |  |
| 17.5 ± 0.6 | - | 25.60 ± 0.09 | - |  |
| 18.1 ± 0.6 | 9.98 ± 0.03 | - | - |  |
| 18.9 ± 0.6 | - | - | 9.40 ± 0.06 |  |
| 19.3 ± 0.6 | - | 25.61 ± 0.09 | - |  |
| 19.7 ± 0.6 | 14.34 ± 0.07 | - | 9.56 ± 0.07 |  |
| 20.0 ± 0.6 | - | 25.17 ± 0.09 | - |  |
| 20.5 ± 0.6 | 15.08 ± 0.07 | - | - |  |
| 21.1 ± 0.6 | - | - | 10.17 ± 0.05 |  |
| 21.5 ± 0.6 | - | 25.29 ± 0.09 | - |  |
| 22.2 ± 0.6 | 8.34 ± 0.04 | - | - |  |
| 23.5 ± 0.5 | - | 25.81 ± 0.08 | - |  |
| 23.5 ± 0.5 | - | 25.60 ± 0.09 | - |  |
| 23.9 ± 0.5 | 11.86 ± 0.06 | - | - |  |
| 23.9 ± 0.5 | 23.99 ± 0.07 | - | - |  |
| 24.8 ± 0.5 | - | 26.94 ± 0.09 | - |  |
| 25.2 ± 0.5 | 13.75 ± 0.07 | - | 9.43 ± 0.05 |  |
| 25.4 + 0.5 | - | 25.29 ± 0.09 | - |  |
| 25.8 ± 0.5 | 8.20 ± 0.07 | - | - |  |
| 26.6 ± 0.5 | - | 25.50 ± 0.09 | - |  |
| 26.8 ± 0.5 | 11.25 ± 0.06 | - | - |  |
| 28.2 ± 0.5 | - | 25.50 ± 0.09 | - |  |
| 28.6 ± 0.5 | 11.22 ± 0.10 | - | - |  |
