# Supplementary material for: Is 70Zn(d,x)67Cu the Best Way to Produce 67Cu for Medical Applications?
Source: Front Med (Lausanne). 2021 Jul 5;8:674617. doi: 10.3389/fmed.2021.674617 (PMC8287065; doi:10.3389/fmed.2021.674617)
Supplement: Supplementary file 3 [file Table_3.docx]

Table S3 - Characteristics of the radionuclides analyzed in this study [2].

| Nuclide | Half-life | E_γ_ (keV) (I_γ_ (%)) | Contributing reaction | Threshold (MeV) |
| --- | --- | --- | --- | --- |
| ^67^Cu | 61.8 h | 184.6 (48.7)  300.2 (0.8) | ^70^Zn(d,n+a)  ^70^Zn(d,n+p+t)  ^70^Zn(d,2n+^3^He)  ^70^Zn(d,n+2d)  ^70^Zn(d,2n+p+d)  ^70^Zn(d,3n+2p) | 0.0  20.0  20.8  24.1  26.4  28.7 |
| ^67^Ga | 78.3 h | 184.6 (21.4)  300.2 (16.6) | ^68^Zn(d,3n) | 14.6 |
| ^61^Cu | 3.339 h | 282.9 (12.2)  373.0 (2.1)  588.6 (1.2)  656.0 (10.8)  908.6 (1.1)  1185.2 (3.7) | ^60^Ni(d,n)  ^61^Ni(d,2n)  ^62^Ni(d,3n) | 0.0  5.4  16.4 |
| ^56^Co | 77.2 d | 846.77 (100)  1037.84 (14.0)  1238.2 (67.6)  1360.215 (4.3)  1771.35 (15.7) | ^58^Ni(d,a)  ^58^Ni(d,p+t)  ^60^Ni(d,2n+a)  ^58^Ni(d,n+^3^He)  ^58^Ni(d,2d)  ^58^Ni(d,n+p+d)  ^61^Ni(d,3n+a)  ^58^Ni(d,2n+2p)  ^60^Ni(d,2t) | 0.0  13.8  14.3  14.5  17.9  20.2  22.4  22.5  26.0 |
| ^58^Co | 70.9 d | 810.8 (99.0) | ^60^Ni(d,a)  ^61^Ni(d,n+a)  ^58^Ni(d,2p)  ^62^Ni(d,2n+a)  ^60^Ni(d,p+t)  ^60^Ni(d,n+^3^He)  ^60^Ni(d,2d)  ^61^Ni(d,d+t)  ^60^Ni(d,n+p+d)  ^61^Ni(d,n+p+t)  ^60^Ni(d,2n+2p)  ^61^Ni(d,2n+^3^He)  ^62^Ni(d,2t)  ^61^Ni(d,n+2d) | 0.0  1.8  1.9  12.7  14.2  15.0  18.3  20.0  20.7  22.3  23.0  23.1  24.4  26.4 |
| ^55^Co | 17.5 h | 411.9 (1.1)  477.2 (20.2)  803.4 (1.9)  931.3 (75.0)  1316.4 (7.1)  1369.7 (2.9)  1408.4 (16.9) | ^58^Ni(d,n+a)  ^58^Ni(d,d+t)  ^58^Ni(d,n+p+t)  ^60^Ni(d,3n+a)  ^58^Ni(d,2n+^3^He)  ^58^Ni(d,n+2d) | 3.7  21.9  24.2  24.8  25.0  28.4 |
| ^57^Co | 271.7 d | 122.06 (85.6)  136.5 (10.7) | ^60^Ni(d,n+a)  ^58^Ni(d,^3^He)  ^58^Ni(d,p+d)  ^61^Ni(d,2n+a)  ^58^Ni(d,n+2p)  ^60^Ni(d,d+t)  ^62^Ni(d,3n+a)  ^61^Ni(d,2t)  ^60^Ni(d,n+p+t)  ^60^Ni(d,2n+^3^He)  ^60^Ni(d,n+2d) | 2.6  2.8  8.5  10.6  10.8  20.8  21.6  22.4  23.1  23.8  27.2 |
| ^48^V | 15.973 d | 983.5 (100.0)  1312.1 (97.5) | ^46^Ti(d,g)  ^47^Ti(d,n)  ^48^Ti(d,2n)  ^49^Ti(d,3n)  ^50^Ti(d,4n) | 0.0  0.0  7.3  15.8  27.2 |
