# Supplementary figures and images for: Is 70Zn(d,x)67Cu the Best Way to Produce 67Cu for Medical Applications?
Source: Front Med (Lausanne). 2021 Jul 5;8:674617. doi: 10.3389/fmed.2021.674617 (PMC8287065; doi:10.3389/fmed.2021.674617)

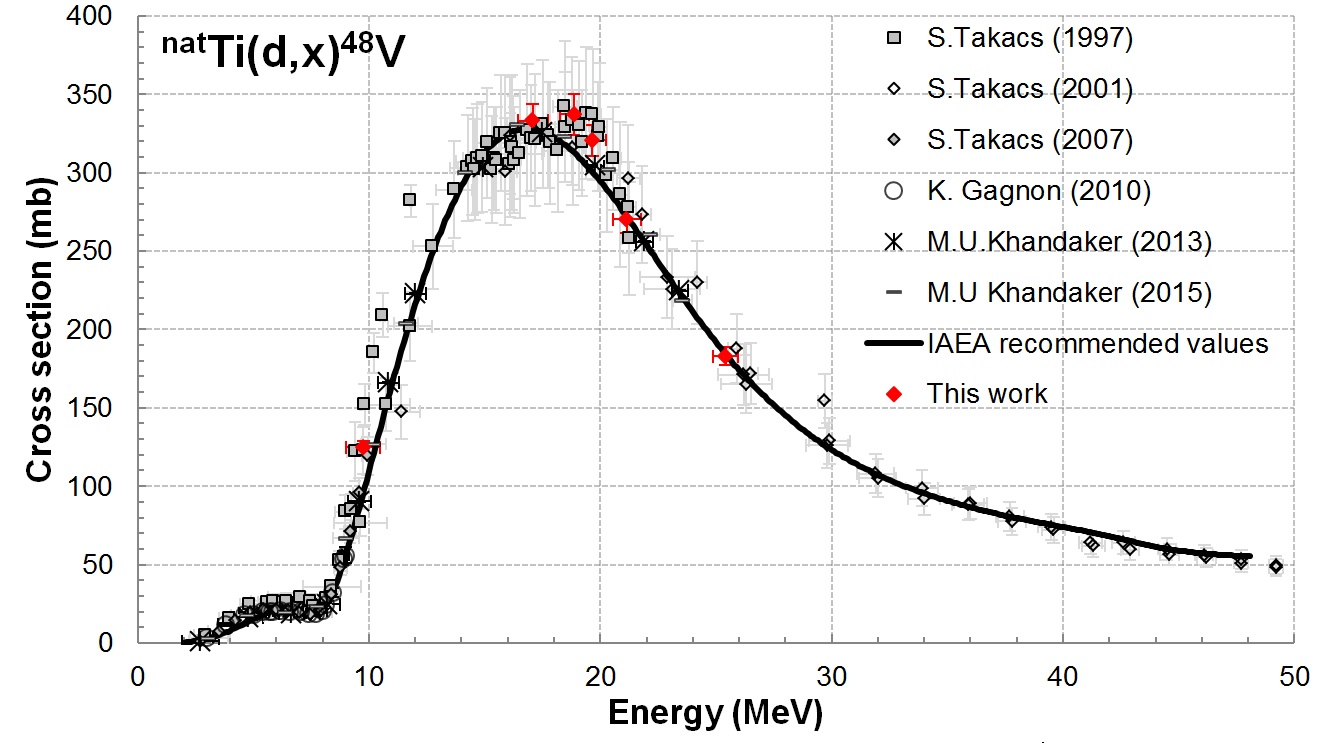

Supplement: Supplementary file 4 [file Image_1.JPEG]

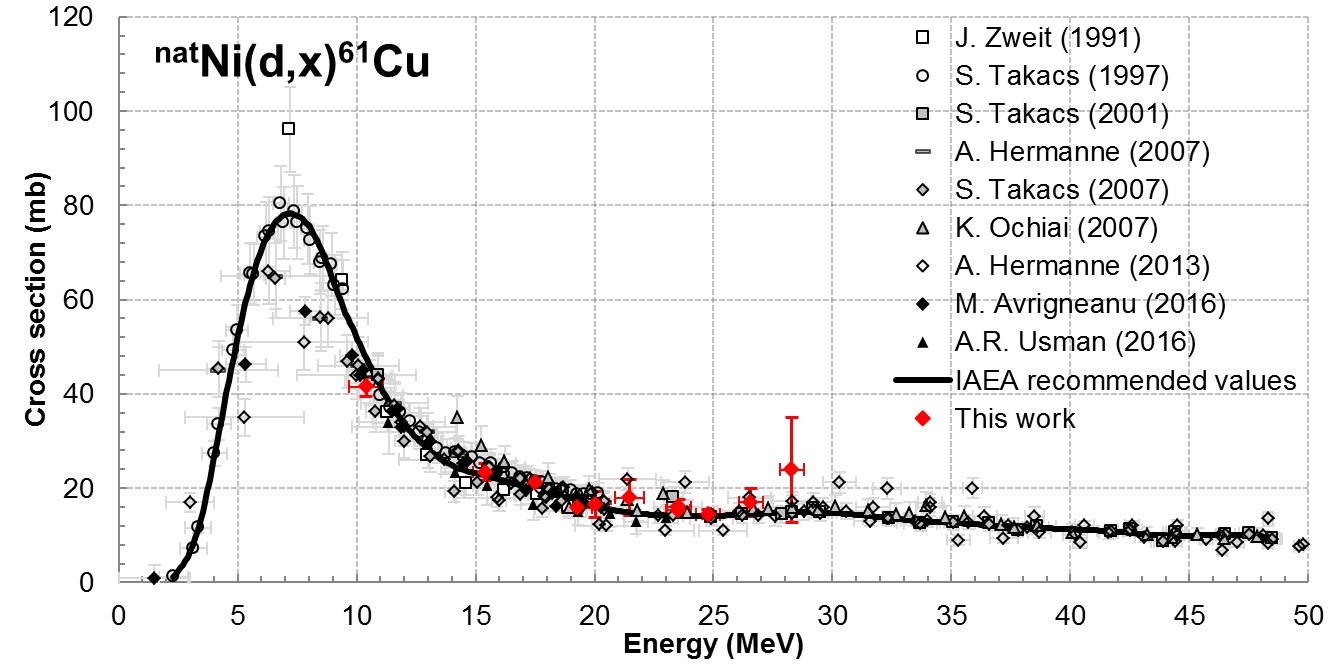

Supplement: Supplementary file 5 [file Image_2.JPEG]
